# The impacts of biodegradable and non-biodegradable microplastic on the performance and microbial community characterization of aerobic granular sludge

**DOI:** 10.3389/fmicb.2024.1389046

**Published:** 2024-05-20

**Authors:** Xiaoying Guo, Xiaohang Ma, Xiangyu Niu, Zhe Li, Qiong Wang, Yi Ma, Shangying Cai, Penghao Li, Honglu Li

**Affiliations:** ^1^School of Ecology and Environment, Zhengzhou University, Zhengzhou, China; ^2^International Joint Laboratory of Environment and Resources of Henan Province, Zhengzhou, China; ^3^College of Water Resources and Environment Engineering, Nanyang Normal University, Nanyang, China; ^4^Ecological Environment Monitoring and Scientific Research Center, Yellow River Basin Ecology and Environment Administration, Ministry of Ecology and Environment, Zhengzhou, China

**Keywords:** aerobic granular sludge, microplastics, polylactic acid, extracellular polymeric substances, microbial community structure

## Abstract

**Introduction:**

Microplastics (MPs), identified as emerging contaminants, have been detected across diverse environmental media. Their enduring presence and small size facilitate the adsorption of organic pollutants and heavy metals, leading to combined pollution effects. MPs also accumulate in the food chain thus pose risks to animals, plants, and human health, garnering significant scholarly attention in recent years. Aerobic granular sludge (AGS) technology emerges as an innovative approach to wastewater treatment. However, the impacts of MPs on the operational efficiency and microbial characteristics of AGS systems has been insufficiently explored.

**Methods:**

This study investigated the effects of varying concentration (10, 50, and 100 mg/L) of biodegradable MPs (Polylactic Acid, PLA) and non-biodegradable MPs (Polyethylene Terephthalate, PET) on the properties of AGS and explored the underlying mechanisms.

**Results and discussions:**

It was discovered that low and medium concentration of MPs (10 and 50 mg/L) showed no significant effects on COD removal by AGS, but high concentration (100 mg/L) of MPs markedly diminished the ability to remove COD of AGS, by blocking most of the nutrient transport channels of AGS. However, both PLA and PE promoted the nitrogen and phosphorus removal ability of AGS, and significantly increased the removal efficiency of total inorganic nitrogen (TIN) and total phosphorus (TP) at stages II and III (*P* < 0.05). High concentration of MPs inhibited the growth of sludge. PET noticeably deteriorate the sedimentation performance of AGS, while 50 mg/L PLA proved to be beneficial to sludge sedimentation at stage II. The addition of MPs promoted the abundance of *Candidatus_Competibacter* and *Acinetobacter* in AGS, thereby promoting the phosphorus removal capacity of AGS. Both 50 mg/L PET and 100 mg/L PLA caused large amount of white *Thiothrix* filamentous bacteria forming on the surface of AGS, leading to deterioration of the sludge settling performance and affecting the normal operation of the reactor. Comparing with PET, AGS proved to be more resistant to PLA, so more attention should be paid to the effect of non-biodegradable MPs on AGS in the future.

## 1 Introduction

Aerobic granular sludge (AGS) technology has attracted widespread attention as an excellent wastewater treatment emerged in the late 1990s (Fu et al., 2022). Compared to Conventional Activated Sludge (CAS), the layered structure of AGS enables it to remove carbonaceous organic matter, nitrogen, phosphorus, heavy metals, and even some difficult-to-degrade aromatic compounds simultaneously. Additionally, its compact particle structure allows for higher biological concentration, faster settling rates, and lower discharge sludge volume (da Silva et al., 2022). AGS technology proved to keep running under the high pollutant loads of influent water and still achieve over 90% removal of chemical oxygen demand (COD), total nitrogen (TN), and total phosphorus (TP) (Nancharaiah et al., 2018). Furthermore, most of the microorganisms in AGS are resistant to toxic compounds in leachate without affecting stability of AGS particle, making AGS a promising technology and replacement for CAS due to its significant advantages for wastewater treatment (Ren et al., 2017).

As a type of emerging pollutant, microplastics (MPs) contamination has been detected in natural water, soil, and atmosphere environment due to the widespread use of synthetic plastic products in worldwide (Kim et al., 2018; Li et al., 2018; Liu et al., 2019; Wang et al., 2019; Winkler et al., 2019). Once released, MPs can hardly be removed from the environment due to of their poor biodegradation properties and small particle sizes. MPs originating from activities such as the laundering of synthetic clothing, utilization of personal care products, and the degradation process of plastic waste, are introduced into municipal wastewater treatment systems. Subsequently, these particles transition into sludge without undergoing complete oxidation and decomposition, ultimately leading to their accumulation within the sludge (Jachimowicz et al., 2022). It was reported that nearly 6.5 × 10^7^ microplastic particles per day would be discharged from WWTPs in Scotland, despite a removal rate of 98% (Murphy et al., 2016), implying that innumerable microplastic particles could be hidden within bioaggregated sludge. Therefore, the activated sludge became a “sink” of plenty MPs in the wastewater treatment system.

Previous studies have shown that MPs could affect the structures, microbial composition, and degradation ability of activated sludge through the biotransformation process (Li et al., 2020; Qin et al., 2020; Zhang and Chen, 2020; Jachimowicz et al., 2022). Li et al. investigated the impact of five types of common MPs (PVC, PP, PE, PS, PES) on the nitrification and denitrification of activated sludge. The results indicated that MPs could inhibit the nitrification process of activated sludge, but the addition of MPs at a concentration of 5000 items/L promoted denitrification in PVC and PES reactors (Li et al., 2020). Another study examined the influence of PES on the nitrification process and microbial community structure in the AGS, demonstrating that PES had a minor inhibitory effect on ammonia nitrogen removal, but increased total nitrogen removal by an average of 5.6%. The high concentration of PES in the AGS system gave rise to the large amounts of PES attached onto the surface of AGS, which impedes the uptake of dissolved oxygen and led to growth of anaerobic microbial and denitrification. PES of low concentration (0.1 g/L) was observed to promote the polysaccharide content of extracellular polymeric substances (EPS), but the high concentration (0.2, 0.5 g/L) of PES led to a decrease in the polysaccharide content of EPS, and affected the stability of AGS particles (Qin et al., 2020). The impact of different concentration (1, 10, and 50 mg/L) of PE MPs on the production of EPS in AGS was assessed. The results showed that the efficiency of pollutant degradation of AGS in was not reduced by PE, and the EPS and alginate content in AGS were enhanced. At a PE loading of 50 mg/L, the alginate content increased from a control of 238.7 ± 4.4 mg/g MLSS to 441.6 ± 13.8 mg/g MLSS (Jachimowicz et al., 2022). Another research reported that PE MPs not only altered the particle structure, settleability, particle size distribution, and extracellular polymer properties of AGS, but also affected the performance of AGS for nitrogen and phosphorus removal ability. Additionally, PE promote the increase of EPS in AGS, thus altering the morphology of AGS and leading to the deterioration of the settling performance (Zheng et al., 2022). Since most MPs are not biodegradable, they could attach to the surface of AGS and accumulate continually, resulting in negative impacts during the treatment of residual sludge (Zhang and Chen, 2020). Therefore, the impacts of MPs on removal ability of conventional pollutants by AGS need to be further investigated and the inherent interacting mechanisms between MPs and AGS need to be elucidated.

It should be noticed that the amount of production and usage of biodegradable plastics have shown an increasing trend in recent year, including polylactic acid (PA), polyhydroxyalkanoate (PHA), polybutylene adipate terephthalate (PBAT), etc. Therefore, a growing number of the biodegradable MPs inevitably transport into environment. Wastewater treatment systems accept the vast majority of both the non-biodegradable and biodegradable MPs from the domestic and industrial wastewater, making it continuously exposed to the impacts of MPs (Carr et al., 2016). Nevertheless, studies concentrating on the effects of the biodegradable MPs on the performance and microbial community characterization of AGS are rarely reported.

In this study, the impacts of the biodegradable MP (PLA) and non-biodegradable MP (PET) on the removal ability and efficiency of conventional pollutants by AGS was investigated via continuous feeding tests that are representative of real wastewater treatment processes. Three stages of experiments (98 days) were conducted, and the effects of PLA and PET on AGS were monitored in terms of removal performances, biomass growth, physicochemical characteristics of granules, secretion of extracellular polymeric substances (EPS), and dynamics of bacterial community compared with a control reactor without MPs. The variations of microbial community in AGS after long-term PLA-MP and PE-MP exposure were also evaluated, which is of crucial importance for biological wastewater treatment. The findings revealed significant differences in the way degradable and non-degradable MPs interact with AGS, with implications for the design and operation of future wastewater treatment systems.

## 2 Materials and methods

### 2.1 AGS and synthetic wastewater composition

The selected original AGS with mean diameters of 2850 μm were cultivated in laboratory for more than 150 days. Prior to the formal MPs experiment, the granular sludge underwent a 2-week acclimatization process to ensure the stabilization of the removal performance of COD, NH_3_-N, and TP of the three reactors. The sludge concentration was consistent for all three reactors, with an average mixed liquor suspended solids (MLSS) concentration of 4070 mg/L. The percentage of mixed liquor volatile suspended solids (MLVSS) was calculated to be 0.69 (MLVSS/MLSS), and the sludge volume index (SVI30) was 41.57 mL/g.

The experimental influent water was synthetic wastewater, and the composition was shown in Table 1 with a COD:N:P ratio of 100:5:1. The total COD (1000 ± 50 mg/L) was provided by CH_3_COONa. The NH_4_^+^-N (50 mg/L) and PO_4_^3–^P (10 mg/L) were afforded by (NH_4_)_2_SO_4_, K_2_HPO_4_-3H_2_O, and KH_2_PO_4_.

**TABLE 1:** Composition of the synthetic wastewater.

Components	Concentration (mg/L)	Trace elements^a^	Concentration (μ g/L)
CH_3_COONa	1282	CoCl_2_⋅6H_2_O	250
(NH_4_)_2_SO_4_	235	CuSO_4_⋅5H_2_O	250
K_2_HPO_4_⋅3H_2_O	15	MnSO_4_⋅H_2_O	250
KH_2_PO_4_	35	NiCl⋅6H_2_O	250
CaCl_2_	46		
MgSO_4_⋅7H_2_O	44		
FeSO_4_⋅7H_2_O	18		
NaHCO_3_	100		

^a^The micronutrient solution added in the synthetic wastewater were used for microbial growth.

### 2.2 Reactor setup and operations

#### 2.2.1 Operating parameter of the reactors

**FIGURE 1:**
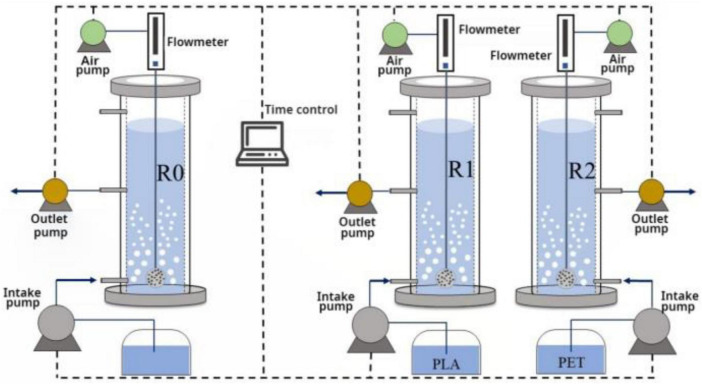
The schematic diagram of the experimental setup.

The experimental set-up consisted of 3 sequencing batch reactors (SBR) with the same working volume of 1.25 L (internal diameter = 5 cm and total height = 75 cm) as shown in Figure 1. The working volume exchange rate was 30%. The SBR was automatically maintained at 25 ± 1°C. The influent was fed into the bottom of the reactor (Cui et al., 2021). Drainage was pumped out of the reactor through the outlet using a drainage pump, and sampling was conducted during the drainage stage. Aeration was achieved by connecting a solenoidal air compressor to the bottom micropore spherical aeration head to introduce air into the reactor. The gas flow rate was controlled using a gas flow meter.

#### 2.2.2 Cycle settings and MPs influent

The three SBR reactors (R0, R1, and R2) were operated under the very same conditions, with each cycle lasting for 4 h cycles. Each cycle included 10 min of anaerobic feeding with 0.375 L wastewater with MPs, 50 min of anerobic, 165 min of aerobic reaction, and 5 min for settling, draining, and standing each. The influent of R1 was added with the biodegradable MPs (Polylactic Acid, PLA) and the influent of R2 was added with the non-biodegradable MPs (Polyethylene Terephthalate, PET) (Figure 1). The entire reaction time was controlled by a time control device. To prevent MPs in the synthetic wastewater from precipitating and aggregating, magnetic stirrers with 200 rpm were used to continuously stir the wastewater to make MPs dispersing relatively evenly in the wastewater.

### 2.3 Experimental stage setting

As shown in Table 2, the experimental period was divided into four stages (stage P, I, II, and III) according to the various MPs loading concentration. The experiment consisted of four stages. The first stage was the preparatory stage (Stage P), which lasted for 2 weeks and involved sludge domestication. During this stage, the influent for all three reactors was identical. The experiment progressed through three stages (Stage I, Stage II, and Stage III). During Stage I, the water inlet conditions of the control reactor (R0) remained unchanged, while the corresponding MPs were added to the experimental reactors (R1, R2). The influent of R1 was added with PLA and that of R2 was added with PET continuously from Stage I to Stage III. The operating parameters of the experimental stage reactors were shown in Table 2. In the recovery stage (Stage R), MPs were no longer added to the experimental reactor influent.

**TABLE 2:** The operation parameters of all reactors during the whole experiments.

Stage	Time (day)	Concentration of MPs (mg/L)		
		R0 (control)	R1 (PLA)	R2 (PET)
Stage P	14	/^a^	/	/
Stage I	30	/	10	10
Stage II	30	/	50	50
Stage III	38	/	100	100
Stage R	8	/	/	/

^a^ “/” indicated that no MPs were added during the certain stage.

### 2.4 Analytical methods

Water samples were regularly collected from the SBR system. During Stage P∼III, samples were taken from the reactor outlet every 2 days. During Stage R, samples were taken every day. Water samples were filtered through a 0.45 μm filter membrane. Chemical oxygen demand (COD), ammonia nitrogen (NH_4_^+^-N), nitrate nitrogen (NO_3_^–^-N), nitrite nitrogen (NO_2_^–^-N), and total phosphorus (TP) were measured in accordance with standard methods (APHA, 2005). Sludge concentration indicators including MLSS and MLVSS, and sludge volume index after 30 (SVI30) and 5 (SVI5) minutes were measured at the end of each stage. Total inorganic nitrogen (TIN) was calculated as the sum of NH_4_^+^-N, NO_3_^–^-N, and NO_2_^–^-N) concentration (Yan et al., 2019). The extracellular polymeric substances (EPS) in the sludge were extracted using a modified heating extraction method (Morgan et al., 1990). The concentration of polysaccharides (PS) in EPS was measured using the phenol-sulfuric acid method with glucose (Kong et al., 2015). The protein content (PN) in EPS was determined using the modified Lowry method with bovine serum protein (Pi et al., 2019). The surface morphology of granular sludge samples was observed using scanning electron microscopy (SEM, FEI Nova Nona 450, USA), and prior to SEM observation, the granular sludge samples should to be stabilized, dried, and sprayed with gold. The significant differences of all results were assessed by statistical analysis, and *p* < 0.05 was regarded as statistically significant in this study.

### 2.5 Microbial community analysis

Sludge samples were taken from each SBR reactor at the end of each stage (initial state, day 30, day 60, day 98) for microbial diversity analysis. Samples were frozen and preserved in an ultra-low temperature refrigerator at −80°C until the DNA extraction. The E.Z.N.A.^®^soil kit (Omega Bio-tek, Norcross, GA, U.S.) was used to perform total DNA extraction from activated sludge. DNA mass concentration and purity were assessed using the Nano Drop 2000. PCR amplification of the V3∼V4 variable regions was conducted sequentially using the general primers 338F (5′- ACTCCTACGGGGAGGCAGCAG-3′) and 806R (5′-GGACTACHVGGGGTWTCTAAT-3′) for bacteria according to previous procedures (Cheng et al., 2021). High-throughput sequencing was performed by Majorbio Bio-Pharm Technology Co. Ltd. (Shanghai, China) using the MiSeq PE300 platform (Illumina, San Diego, USA) according to the manufacturer’s instructions. Quality control of the raw sequences was performed using fastp software (Li et al., 2015), splicing was done using FLASH software (Edgar, 2013), and OUT clustering of sequences using 97% similarity was performed using UPARSE software (Wang et al., 2007). The PDR classifier (Avella et al., 2010) was used to annotate species classification for each sequence by comparing it to the Silva 16S rRNA database (version 138) with a comparison threshold of 70%.

## 3 Results and discussions

### 3.1 Characterization of MPs-AGS system

#### 3.1.1 Effect of MPs on AGS surface morphology

**FIGURE 2:**
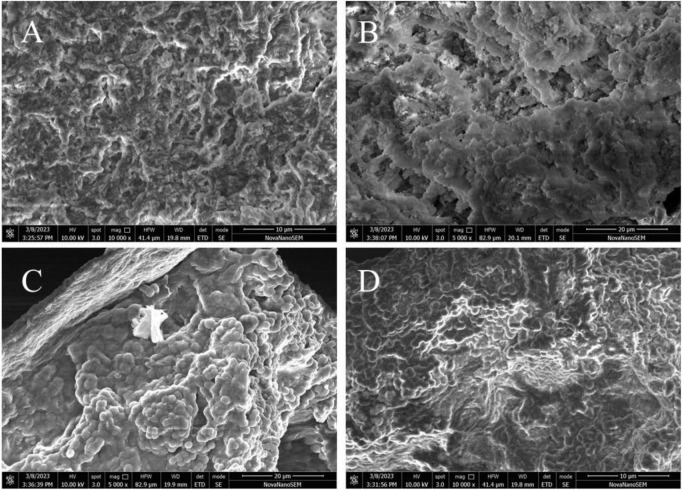
SEM images of sludge in the reactor at the end of stage P **(A)**, at the end of stage III in R0 **(B)**, R1 **(C)** and R2 **(D)**.

The surface morphology of the granular sludge in the three reactors was observed using electron scanning microscopy at the end of Stage P and at the end of Stage III to determine the retention status of MPs inside the AGS. Comparison of Figures 2A, B shows that the AGS becomes looser in sludge structure after approximately 90 days of operation, while still retaining numerous void channels that facilitate nutrient delivery to the interior part of granules (Cydzik-Kwiatkowska et al., 2013). Figures 2C, D show that the sludge surface is compact and has a small number of channels, making nutrient transport difficult. This is due to the R1 and R2 reactors losing their ability to remove COD at Stage III. Furthermore, Figure 2C showed that AGS was able to trap PLA on its surface, demonstrating its effectiveness in removing not only conventional pollutants but also this emerging type of pollutant from wastewater.

#### 3.1.2 Impact of MPs on biomass retention and granular settleability

**FIGURE 3:**
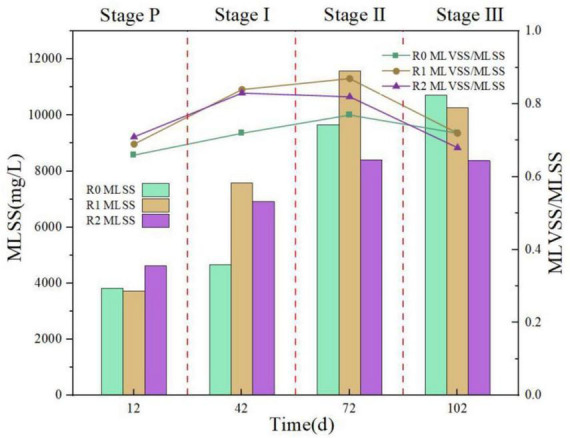
Sludge concentration in the reactor at different stages.

Sludge concentration is a crucial indicator of biomass in the SBR reactor, as depicted in Figure 3. On day 12 (end of Stage P), the sludge concentration of R0, R1 and R2 reactors were 3831 mg/L, 3732 mg/L, and 4635 mg/L, respectively. The MLVSS/MLSS ratios were 0.66, 0.69, and 0.71. Respectively, at this point, the sludge volume and biomass concentration of the three reactors were essentially the same and had stabilized at this level. After adding 10 mg/L of PLA and PET to R1 and R2 reactors for 30 days (end of Stage I), the sludge concentration in R1 and R2 reactors was significantly higher than that in R0 reactor. Additionally, the values of MLVSS/MLSS also increased, indicating that low concentration of MPs promoted the growth of sludge. At the end of Stage II (day 72), the sludge concentration in the R1 reactor continued to rise remarkably (48.3%) and the sludge concentration was higher than that of R0. However, R2 displayed a much lower increase (18.5%) in sludge concentration. The MLVSS/MLSS value of R2 was also decreasing. It indicated that PLA (50 mg/L) enhanced on the sludge growth of R1, while PET (50 mg/L) showed little positive effects on the increase of sludge concentration, resulting in a lower sludge concentration in R2 compared to R0 by the end of the stage II. At the end of Stage III, after 103 days, only the sludge concentration in R0 reactor had increased. In contrast, the sludge concentration in both R1 and R2 reactors had decreased, and the MLVSS/MLSS values had decreased more rapidly than those in R0 reactor. These results suggest that the growth of sludge is inhibited and the biomass is decreasing at a microplastic concentration of 100 mg/L.

Overall, the sludge showed greater tolerance to the biodegradable PLA. This may be due to the sludge in the reactor degraded part of the PLA, making it a carbon source for microbial growth (Song et al., 2022). However, when the concentration of PLA was increased to 100 mg/L, its degradation slowed down, resulting in a similar phenomenon as observed for the non-biodegradable PET at 50 mg/L.

#### 3.1.3 Effect of MPs on sludge settling performance

**FIGURE 4:**
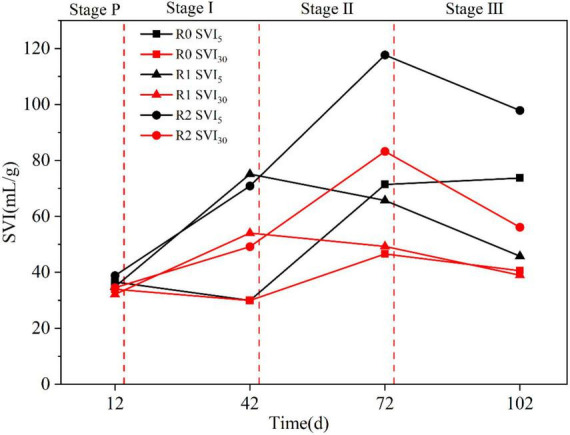
Sludge settling performance of different reactors in stages I∼III.

The variations of sludge settling performance among R0, R1, and R2 reactors during Stage P∼III were displayed in Figure 4. In Stage P, the SVI_5_ and SVI_30_ of all three reactors were similar. At the end of Stage I, the SVI_5_ and SVI_30_ of the R0 reactor decreased, while those of the R1 and R2 reactors increased. This suggests that 10 mg/L MPs promoted sludge growth, but the newly grown sludge was mainly flocculated sludge with poor settling performance. At the end of Stage II, the SVI_5_ and SVI_30_ of the R1 reactor decreased, while there was a significant increase in the R2 reactor. Despite the relatively intact particle morphology of sludge particles within the R2 reactor, it exhibited poor settling performance. The white flocs present on the surface of the sludge were also widely distributed within the reactor. These flocs proved difficult to settle during the settling process, resulting in an elevated SVI5 in the R2 reactor. This may be attributed to a change in the EPS nature of AGS, specifically an increase in hydrophobicity (Zheng et al., 2022).

In the Stage II and III, a continuing reduction of SVI_5_ and SVI_30_ was observed with R1, suggesting that the presence of PLA (50, 100 mg/L) contributed to the granulation of sludge and its settling properties. At Stage II, the granular sludge had started to adapt to PLA, with an onset of improvement in settling performance. Differently, it was not until Stage III that both SVI_5_ and SVI_30_ parameters of R2 began to decrease, suggesting that PET of 50 mg/L adversely affect the growth and settling performance of the sludge. At the same MPs concentration, granular sludge exhibits poorer adaptability to the non-biodegradable PET than the biodegradable PLA, requiring a longer time to adjust to PET than PLA. Geng et al. (2021) also found that without adding MPs, the granules lost their morphological integrity, leading to a deterioration of settling properties during prolonged operation.

#### 3.1.4 Effect of MPs on the EPS

**FIGURE 5:**
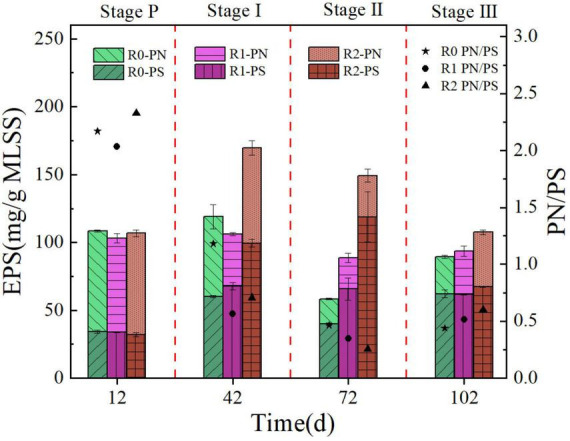
Sludge EPS content of R0, R1 and R2 reactors at Stage P∼III.

EPS is a macromolecular substance distributed on the cell surface that promotes microbial aggregation, is an important part of microbial aggregates, and plays a crucial role in resisting toxic and harmful substances. The experiments measured the EPS of the three reactors at Stage P∼III, as shown in Figure 5. At the end of Stage P, the content of EPS and PN/PS of the three reactors were similar. During Stages I-III, the EPS content in the R0 reactor fluctuated, while the R1 reactor remained more stable. Both R0 and R1 reactors showed their lowest values during Stage II, with 58.38 mg/g MLSS and 88.66 mg/g MLSS, respectively. The EPS content in the R2 reactor increased significantly during Stage I, but then decreased continuously, reaching its lowest value of 107.56 mg/g MLSS during Stage III. Research has demonstrated that PE an stimulate the production of EPS by AGS (Jachimowicz et al., 2022; Zheng et al., 2022). Similarly, PET, another non-biodegradable MPs, was found to have a promoting effect on the production of EPS by AGS in experimental results.

During domestication in Stage P, the PN/PS values in the three reactors ranged from 2.03 to 2.33. As the reactors operated, the PN/PS value in the R0 reactor continuously declining. At the end of Stage III, the PN/PS values in the R1 and R2 reactors reached their lowest points in Stage II, measuring 0.35 and 0.26, respectively. Subsequently, in Stage III, the values increased to 0.52 and 0.60. The PN/PS values are indicative of the aggregate performance of microorganisms in both flocculent and aerobic granular sludge, with higher ratios indicating better sludge aggregation performance (Ni et al., 2009; Zhu et al., 2012). The results of sludge morphology in Figure 2 are consistent with the indication that the particle stability of the aerobic granular sludge in the R0 reactor is decreasing over time. In Stage III, the values of PN/PS in the R1 and R2 reactors increased compared to Stage II. This suggests that a high concentration of MPs may stimulate the AGS, prompting the sludge to granulate. Future research should consider the effect of higher concentration of MPs on sludge granulation.

### 3.2 Effect of MPs on the performance of AGS system

#### 3.2.1 Effect of MPs on COD removal by AGS

**FIGURE 6:**
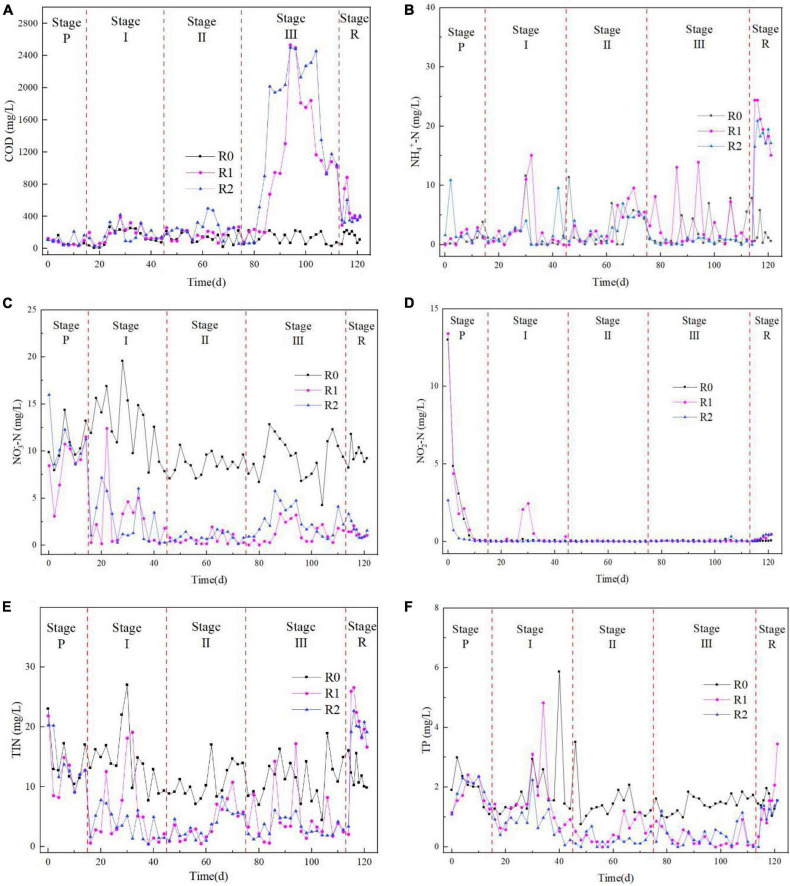
The variation of COD **(A)**, NH_4_^+^-N **(B)**, NO_3_^–^-N **(C)**, NO_2_^–^-N **(D)**, TIN **(E)**, and TP **(F)** during the whole experiment.

After a 2-week acclimation period at Stage P, the activated sludge in all three reactors exhibited consistent COD removal performance, with no significant differences observed between the R0 reactor and the R1 and R2 reactors. As shown in Figure 6A, at Stage I, there were no significant differences in effluent COD concentration between the R0 reactor and the R1 and R2 reactors, indicating that the addition of 10 mg/L of MPs had no impact on COD removal by AGS. However, at Stage II, significant differences in effluent COD concentration between the R0 reactor and the R1 and R2 reactors were observed, indicating that the addition of 50 mg/L of MPs had a significant impact on COD removal by AGS. During Stage III, as the microplastic concentration increased to 100 mg/L, the AGS’s ability to remove COD rapidly diminished after 1 week. The effluent COD concentration in the R1 and R2 reactors increased to over 2000 mg/L, indicating that at high concentration (100 mg/L), both PLA and PET can cause a loss of AGS’s ability to remove COD. The increase in effluent COD concentration beyond the influent concentration may be due to the massive death of the sludge. However, as the sludge adapted, the effluent COD concentration recovered to approximately 1000 mg/L toward the end of the stage. During the recovery stage of Stage R, the COD concentration of R1 and R2 reactors’ effluent did not recover to the same level as R0 reactor in the short term. This indicates that even if MPs are removed from the influent, the impact of MPs on AGS to remove COD still exists in the short term.

#### 3.2.2 Effect of MPs on NH_4_^+^-N removal by AGS

The NH_4_^+^-N removal performance of the activated sludge in the three reactors was comparable after 2 weeks of domestication in Stage P with no significant difference. Similarly, in Stage I and Stage II, there was no significant difference in the effluent NH_4_^+^-N concentration among the three reactors. In Stage III, there was no significant difference in NH_4_^+^-N removal between R1 and R0. However, there was a significant difference between R2 and R0, indicating that the addition of PLA did not significantly affect the ability of AGS to remove NH_4_^+^-N (Figure 6B). Additionally, PET inhibited the removal of NH_4_^+^-N by the AGS only at high concentration. During the recovery phase Stage R, the concentration of NH_4_^+^-N in the effluent water of the R1 and R2 reactors increased rapidly. This may be due to AGS adapting to the high concentration of MPs in the environment. However, the sudden disappearance of MPs in the influent water led to changes in the influent water environment, which affected the removal of NH_4_^+^-N by AGS.

#### 3.2.3 Effect of MPs on NO_3_^–^-N removal by AGS

The NO_3_^–^-N removal performance of the activated sludge in the three reactors converged after 2 weeks of domestication in stage P (Figure 6C). The variability between R0 and R1 (*P* = 0.048) was due to fluctuations in the pre-stage of the R1 reactor, but stage P had already converged in the NO_3_^–^-N concentration in the effluent at the end of the stage. The addition of 10 mg/L MPs significantly enhanced the AGS’s capacity for NO_3_^–^-N removal. Consequently, NO_3_^–^-N concentrations in the effluents from reactors R1 and R2 were markedly lower than those in the control reactor (R0) (Figure 6C). With the increase of MPs concentration, the NO_3_^–^-N content in the effluent of R1 and R2 reactors still lower than R0. During the stage R, the NO_3_^–^-N concentration in the effluent from R1 and R2 reactors remained low after MPs were no longer added to the influent. It was shown that 10 mg/L of MPs inhibited the nitrification reaction of AGS, but then the inhibition of AGS nitrification by MPs disappeared when the microplastic concentration was increased to 100 mg/L (Wang et al., 2021). The addition of MPs resulted in lower NO_3_^–^-N concentration in the effluent, but there was no significant change in NH_4_^+^-N. The results indicated that MPs exerted no significant influence on the nitrification process of AGS. Furthermore, it was observed that the presence of MPs enhanced the capability of AGS to eliminate NO_3_^–^-N. Notably, this enhancement persisted even after the cessation of MPs in the influent, suggesting a lasting effect on the AGS’s denitrification efficiency.

#### 3.2.4 Effect of MPs on NO_2_^–^-N removal by AGS

The NO_2_^–^-N removal performance of the activated sludge in the three reactors converged after 2 weeks of domestication in the stage P (Figure 6D). There was no difference between R0 reactor and R1 and R2 in stage I, stage II and stage III, indicating that the addition of MPs had less effect on the anaerobic denitrification process of AGS. In stage R, the NO_2_^–^-N concentration of the effluent from R1 and R2 reactors increased and showed differences from R0 reactor.

Combining the effects of MPs on the removal of NH_4_^+^-N, NO_3_^–^-N and NO_2_^–^-N by AGS, the NO_3_^–^-N concentration in the effluent of R1 and R2 decreased rapidly after the addition of MPs from the influent of the experimental stage, and NH_4_^+^-N in the effluent of R1 and R2 increased rapidly in the recovery stage, and NO_2_^–^-N also increased in the recovery stage, which indicated that the addition of MPs had a positive effect on the denitrification of AGS denitrification reaction by adding MPs.

#### 3.2.5 Effect of MPs on total inorganic nitrogen removal by AGS

Total Inorganic Nitrogen (TIN) is the sum of NH_4_^+^-N, NO_3_^–^-N and NO_2_^–^-N in the effluent. In Stage P, there was no difference in effluent TIN among the three reactors; the rapid decrease in TIN in R1 and R2 reactors in Stage I was caused by a rapid decrease in NO_3_^–^-N and was maintained at a lower level than in R0 reactor in Stages II and III; in Stage R, the effluent TIN of R1 and R2 reactors was caused by the rapid increase in NH_4_^+^-N. From the effluent TIN, the addition of MPs promotes the removal of nitrogen from the effluent, while the sudden disappearance of MPs from the influent results in a rapid decrease in nitrogen removal capacity.

#### 3.2.6 Effect of MPs on TP removal by AGS

In Stage P, the TP removal performance of activated sludge in the three reactors converged after 2 weeks of domestication. In Stage I, there was no significant difference between R1 and R0, while there was a significant difference between R2 and R0. Based on Figure 6E, the addition of only 10 mg/L of PET MPs in the R2 reactor resulted in increased TP removal. As the concentration of MPs increased to 50 mg/L and 100 mg/L, both PLA and PET MPs had a similar effect on promoting TP removal. During the Stage R phase, Figure 6F showed a rapid increase in TP levels in the effluent to the R0 level after MPs were no longer added to the R1 and R2 influent. Additionally, there was a tendency for TP levels in the effluent from the R1 reactor to increase.

### 3.3 Effect of MPs on AGS microbial communities

Microbial diversity was analyzed through sequencing of 16S rRNA gene sequences. Sludge samples at the end of each stage were taken for sequencing. At the end of Stage P, the sludge in the three reactors was completely consistent so that the sludge at this time was used as a control for the original sludge, named Stage_P_YS. The sludge from the three reactors in Stage I to III were then named Stage I R0, Stage I R1, Stage I R2, Stage II R0, Stage II R1, Stage II R2, Stage III R0, Stage III R1, and Stage III R2. The study analyzed the effects of different concentration of MPs on the biological communities of AGS at different stages.

**FIGURE 7:**
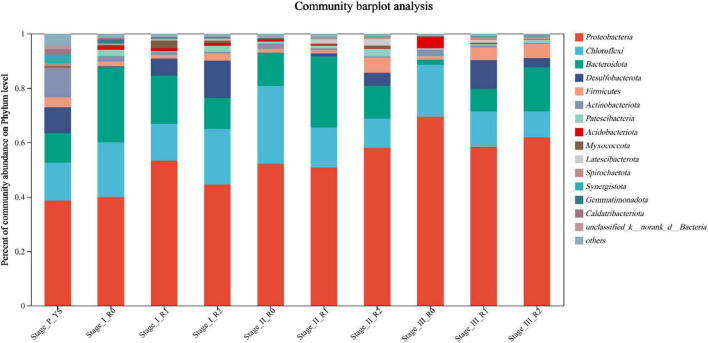
Microbial community composition at the activated sludge phylum level at the end of Stage I∼III.

Abundance change analysis was conducted on the phylum with relative abundance greater than 1%, while the remaining bacteria were categorized as ‘other’ at the phylum level. Figure 7 shows that the phylum Proteobacteria was the dominant phylum in the original control sludge. The abundance of Proteobacteria in the R0 reactor increased with the operation of the reactor, from 38.7% in Stage P to 69.5% in Stage III. The R1 and R2 reactors also showed an overall increasing trend. Proteobacteria and Bacteroidota are the dominant phyla when sodium acetate is used as a single carbon source. One of the primary functions of the Proteobacteria phylum is the degradation of COD (Chen et al., 2023). Even though the abundance of Proteobacteria in R1 and R2 did not decrease during Stage III, the efficiency of COD removal by both R1 and R2 was significantly reduced. According to the study, high concentration of MPs obstructed the nutrient transport pathway in the AGS, resulting in a considerable reduction in COD removal by the AGS.

After the addition of MPs, the abundance of Chloroflexi in the R1 and R2 reactors was consistently lower than in the R0 reactor. This may be due to the reduced light intensity in the reactors caused by the MPs. Chloroflexi is capable of photosynthetic autotrophy (Shih et al., 2017), which could explain its higher abundance in the R0 reactor. Additionally, the abundance of the Desulfobacterota phylum in R0 reactor during Stages I to III was 0.36%, 0.03%, and 0.09%, respectively. Nevertheless, the abundance of Desulfobacterota in the R1 reactor was 6.33% at Stage I, 1.14% at Stage II, and 10.51% at Stage III. The abundance of Desulfobacterota in R2 reactors during Stages I to III was 13.71%, 4.77%, and 3.40%, respectively. The results demonstrated that the abundance of the Desulfobacterota in both R1 and R2 were much higher than that in R0. According to the research (Liu et al., 2021), Desulfobacterota is associated with sulfur and phosphorus cycling in the water column. Therefore, phosphorus removal efficiency was promoted by PLA and PE in R1 and R2 reactors, compared to R0 reactor without MPs.

**FIGURE 8:**
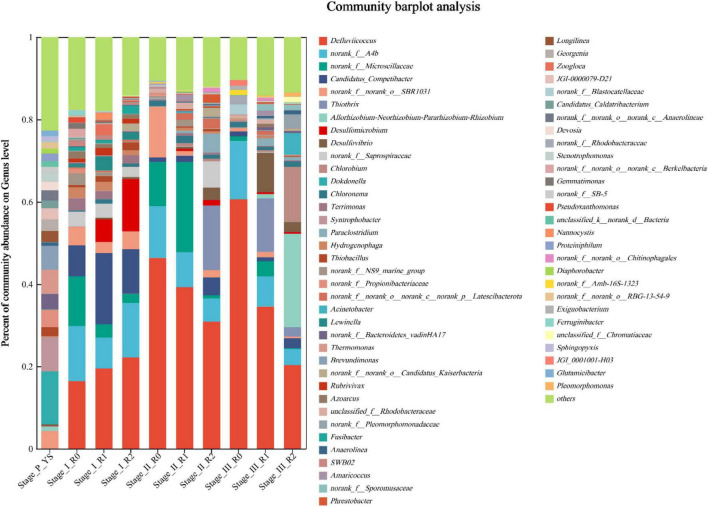
Microbial community composition of activated sludge genus level at the end of Stage I∼III.

At the genus level, we further screened genera with a relative abundance greater than 1% for analysis. As depicted in Figure 8, *Defluviicoccus* was the most dominant group. *Defluviicoccus* is a genus within the phylum of Proteobacteria, known for its preference for acetate (Chen et al., 2021). Since the simulated wastewater had a single carbon source of sodium acetate, *Defluviicoccus* became the dominant group. *Candidatus_Competibacter* is a glycoconjugate. It plays a crucial role in phosphorus removal and supports the anoxic activity of polyphosphate bacteria by contributing to the reduction of nitrate to nitrite (Xia et al., 2018). Its abundance was 7.53%, 17.29%, and 10.84% in Stage I R0, R1, and R2 reactors, respectively, 1.31%, 1.58%, and 4.35% in Stage II, and 1.15%, 0.96%, and 2.40% in Stage III. *Acinetobacter*, the first discovered polyphosphate bacterium, had a relative abundance of 0.09% in Stage_P_YS. In subsequent stages, the abundance of *Acinetobacter* remained consistently at 0.00% in the R0 reactor. In Stages I to III of the R1 reactor, the abundance of *Acinetobacter* was 0.01%, 0.39%, and 0.11%, respectively. In Stages I to III of the R2 reactor, the abundance of *Acinetobacter* was 0.06%, 0.15%, and 5.35%, respectively. After the addition of MPs to the influent water of R1 and R2 reactors, the abundance of *Acinetobacter* was higher than that of R0 reactor, which corresponded to the concentration of TP in the effluent water, as shown in Figure 6F. In addition, MPs facilitated phosphorus removal by AGS.

*Desulfomicrobium* and *Desulfovibrio* are genera within the phylum Desulfobacterota, which are involved in the cycling of sulfur and phosphorus. In Stage P, the abundance of *Desulfomicrobium* was 0.03% and the abundance of *Desulfovibrio* was 4.75%. In the R0 reactor, the abundance of *Desulfomicrobium* and *Desulfovibrio* remained consistently at 0.00% from Stage I to III. However, in the R1 reactor, the abundance of *Desulfomicrobium* was 5.51%, 0.52%, and 0.45% at Stage I, II and III, respectively. The abundance of *Desulfovibrio* was 0.34%, 0.48%, and 9.60% at Stage I, II and III, respectively. In the R2 reactor, the abundance of *Desulfomicrobium* was 12.69%, 1.32%, and 0.38%, and the abundance of *Desulfovibrio* was 0.51%, 3.04%, and 2.50% in Stage I, II and III, respectively. The results indicated that the presence of PLA and PE at different concentration affected the growth and transformation of *Desulfomicrobium* and *Desulfovibrio*. Specifically, a low concentration of 10 mg/L promoted the growth of *Desulfomicrobium* with *Desulfovibrio*, while medium (50 mg/L) and high (100 mg/L) concentration led to the transformation of *Desulfomicrobium* to *Desulfovibrio*.

*Thiothrix* is a typical filamentous bacterium. Filamentous *Thiothrix* was found in abundance of 15.65%, 12.95%, and 2.23% in the R2 reactor of Stage II and in the R1 and R2 reactors of Stage III, respectively. These abundance correspond to the large amount of white filaments attached to the surface of the granular sludge in the corresponding stages. The abundance of *Thiothrix* can interfere with oxygen transfer, during wastewater treatment processes, leading to sludge expansion (He et al., 2021). Additionally, an increase in *Thiothrix* abundance can cause deterioration in sludge settling performance within the reactor.

## 4 Conclusion

This study investigated the influences of PLA and PET on settling performance, EPS, the removal performance of conventional pollutants of AGS, as well as the variations in the AGS microbial community. PLA of 10 and 50 mg/L promoted sludge growth, while PET inhibited the growth of AGS at 50 and 100 mg/L. PLA facilitated the settling performance at 50 and 100 mg/L, while PET severely impaired settling performance due to the formation of white filamentous fungi in the surface of AGS. PLA displayed minor impacts on the production of EPS by AGS, but PET (10 mg/L) promoted the generation of EPS. Both PLA and PET of high concentration led to complete corruption of COD removal capability by AGS. However, the MPs showed promoting effects on AGS denitrification and phosphorus removal capabilities. The absence of MPs in influent immediately weakened the denitrification and phosphorus removal capacities of AGS. MPs altered the microbial community composition of AGS, with Proteobacteria being the dominant phylum. In Stage III, despite the abundance of Proteobacteria of R1 and R2, COD removal capability declined substantially, indicating that MPs affect the channels for transporting nutrients in AGS. The incorporation of MPs promoted the proliferation of *Candidatus_Competibacter* and *Acinetobacter*, thereby enhancing AGS phosphorus removal capabilities. 10 mg/L MPs promotes the growth of *Desulfomicrobium* and *Desulfovibrio*, while higher concentration (50 mg/L and 100 mg/L) induce a transition from *Desulfomicrobium* to *Desulfovibrio*. The manifestation of white filamentous material on particle surfaces was identified as sulfur bacteria (*Thiothrix*), with a pronounced increase observed at 50 mg/L of PET and 100 mg/L of PLA, leading to compromised sludge settling performance and perturbation in the normal functioning of the reactor. The study highlights the complex interactions between microplastics, microbial communities, and wastewater treatment processes. Future research could pay attention to the long-term effects of MPs on AGS and explore the mechanisms through which MPs influence microbial community dynamics and their metabolic pathways.

## Data availability statement

The original contributions presented in the study are included in the article, further inquiries can be directed to the corresponding author.

## Author contributions

XG: Funding acquisition, Writing – original draft, Writing – review and editing, Conceptualization, Data curation, Formal analysis, Investigation, Methodology, Project administration, Resources, Software, Supervision, Validation, Visualization. XM: Investigation, Methodology, Writing – review and editing. XN: Writing – original draft, Methodology. ZL: Investigation, Writing – review and editing. QW: Investigation, Writing – review and editing. YM: Visualization, Writing – review and editing. PL: Data curation, Investigation, Writing – original draft. HL: Resources, Writing – review and editing, Supervision. SC: Investigation, Writing – review and editing.
